# ONT in Clinical Diagnostics of Repeat Expansion Disorders: Detection and Reporting Challenges

**DOI:** 10.3390/ijms26062725

**Published:** 2025-03-18

**Authors:** Ludmila Kaplun, Greice Krautz-Peterson, Nir Neerman, Yocheved Schindler, Elinor Dehan, Claudia S. Huettner, Brett K. Baumgartner, Christine Stanley, Alexander Kaplun

**Affiliations:** Variantyx Inc., Framingham, MA 01701, USA; greice.krautz@variantyx.com (G.K.-P.); brett.baumgartner@variantyx.com (B.K.B.);

**Keywords:** ONT, long reads, genetic testing, ataxia, dementia, WGS, repeat expansion, Nanopore

## Abstract

While whole-genome sequencing (WGS) using short-read technology has become a standard diagnostic test, this technology has limitations in analyzing certain genomic regions, particularly short tandem repeats (STRs). These repetitive sequences are associated with over 50 diseases, primarily affecting neurological function, including Huntington disease, frontotemporal dementia, and Friedreich’s ataxia. We analyzed 2689 cases with movement disorders and dementia-related phenotypes processed at Variantyx in 2023–2024 using a two-tiered approach, with an initial short-read WGS followed by ONT long-read sequencing (when necessary) for variant characterization. Of the 2038 cases (75.8%) with clinically relevant genetic variants, 327 (16.0%) required additional long-read analysis. STR variants were reported in 338 cases (16.6% of positive cases), with approximately half requiring long-read sequencing for definitive classification. The combined approach enabled the precise determination of repeat length, composition, somatic mosaicism, and methylation status. Notable advantages included the detection of complex repeat structures in several genes such as *RFC1*, *FGF14*, and *FXN*, where long-read sequencing allowed to determine somatic repeat unit variations and accurate allele phasing. Further studies are needed to establish technology-specific guidelines for the standardized interpretation of long-read sequencing data for the clinical diagnostics of repeat expansion disorders.

## 1. Introduction

Reduction in the cost of next-generation sequencing (NGS) in recent years have paved the way for a shift in the paradigm of clinical genetic testing, allowing for comprehensive whole-genome sequencing (WGS)-based technology to be used in first-line diagnostic tests [1,2,3]. Currently, the standard approach to clinical diagnostic WGS relies on short-read sequencing due to the highly accurate calling of most types of genetic variants, reasonable costs of the required equipment and consumables, and availability of lab automation. Despite the many advantages of this approach, short-read sequencing technologies still have significant intrinsic limitations that have not been fully addressed, even with the wide array of existing bioinformatic analytical tools [4,5,6,7,8]. Among these limitations are problematic areas such as nonunique genomic regions and tandem repeats, which include short tandem repeats of few base pairs (STRs), longer variable number tandem repeats (VNTRs), and centromere/telomere microsatellites [9,10]. Those shortcomings are due to read length constraints that prevent the spanning of larger variants and may cause ambiguous mapping within repetitive genomic regions [4,6,7]. It has been demonstrated that such genomic regions are best analyzed with long-read sequencing technologies that have the ability to resolve both the overall length and the actual sequence of these low complexity regions. Moreover, long-read sequencing adds information on DNA methylation status, which is critical for the interpretation of some genetic variants [11,12,13,14,15].

Pathogenic STR expansions are one of the major types of genetic aberrations associated with a range of phenotypic abnormalities, primarily with neurological symptoms (such as ataxia, epilepsy, and cognitive impairment) and, in some conditions, also with physical manifestations. Over 50 diseases, including Huntington disease, frontotemporal dementia, Friedreich’s ataxia, spinocerebellar ataxias, cerebellar ataxia, neuropathy, and vestibular areflexia syndrome (CANVAS), etc., are currently associated with STR expansions, and this number grows annually with increased awareness and technological advances [12,16,17,18,19,20,21,22,23,24]. Pathogenic STRs vary by location, composition of the repeat unit, and size of the expansions [9,25,26,27,28,29]. For most STRs, repeat size ranges are defined as normal, mutable normal, premutation, reduced penetrance, or full penetrance alleles. STRs are highly heterogeneous variants, where not only the repeat unit count but also the composition of the repeat loci may differ between individuals, whether healthy or affected. While some pathogenic STR expansions just represent longer stretches of the same repeat units that are found in the unaffected population, other STR regions have pathogenic repeat units which differ from the benign unit type. Such an expansion might completely replace the benign units or be embedded among them and might even be composed of different stretches of various repeats, some of which have yet to be decidedly classified as benign or pathogenic. In addition, the somatic variability of the repeat length and composition is also a known feature of some STR regions [30,31,32]. In some conditions, the composition of the expanded repeat regions may influence the age of onset, penetrance, and severity of symptoms, with interrupting sequences acting as stabilizing factor that affects the extent of somatic heterogeneity in the individual and repeat length expansion/contraction in transfer between generations [12,29,30,33,34,35,36,37,38,39,40].

In cases of recessive disorders associated with expanded STRs, both the detection of expanded allele/s and the specific size of each allele are critical for the detection of the diagnostic variant, although these data might not always be apparent in some testing approaches. Moreover, in some recessive conditions, the combination of a pathogenic STR allele and a pathogenic non-STR variant on the other allele might also lead to a phenotype. Therefore, the ability to perform highly accurate analyses of STR repeat length, allele repeat unit composition, and allele phasing is paramount for an accurate diagnosis. However, pathogenic STR repeat expansions often stretch beyond the dimensions of the short sequencing reads, which are typically limited to 150 bp, making the determined variant length only predictable and the exact region composition unattainable.

The vast majority of clinical diagnostic laboratories use PCR, repeat-primed PCR, or Southern blot to evaluate STR expansion length and mosaic variability. Of these techniques, only repeat-primed PCR may assist with detection of the repeat region composition. These methods require specific assays for each tested STR, have high DNA input requirements, and are expensive, particularly when multiple STRs need to be examined [39].

Here we demonstrate that the combination of short-read-based WGS with ONT long-read sequencing for the detection of STR expansions significantly improves the diagnostic performance of clinical short-read WGS-based genetic testing for neurological disorders. Such a combined approach allows for the differentiation of pathogenic, premutation, intermediate, and benign repeat expansions even at a longer range, as well as the determination of repeat loci compositions and methylation status, where applicable. Moreover, since this approach is not targeted, it intrinsically includes all STRs and, upon the discovery of novel disease-causing STR expansions, the existing sequencing data can be reanalyzed to examine those additional regions. We also discuss challenges in interpretation and clinical reporting of NGS-based test results due to differences between this technique and the conventional methods that form the basis of the current clinical interpretation guidelines.

## 2. Results

The typical processing routine of most clinical genetic tests at Variantyx utilizes a two-tiered approach. First, every patient sample undergoes a short-read-based WGS. While small sequence changes, longer structural variants, mitochondrial variants, and some shorter STR expansions can be fully analyzed with short-read data alone, other variants require further characterization to determine their pathogenicity. Such variants mostly include longer STR expansions, structural variants in low-complexity regions, and recessive variants of any kind where pathogenicity detection depends on the ability to reliably haplotype the alleles. When such potentially reportable variants are detected with short-read WGS, the sample undergoes additional WGS using ONT long-read sequencing [41].

To assess the diagnostic value of long-read sequencing as a complementary approach to the short-read WGS testing of neurological disorders, we performed a retrospective analysis of 2689 cases with movement disorders and dementia-related phenotypes processed at Variantyx in 2023 and 2024.

In 2038 cases (75.8%) one or more clinically relevant genetic variants were reported, including STR expansions and other variant types such as small sequence changes, structural variants, and mitochondrial variants (see Table 1). Cases where STRs were included in the clinical report alone or in combination with another variant(s) are presented in the ‘STR expansion’ category. Cases without reportable variants (651 cases, 24.2%) are not included in this table.

Out of these 2038 cases, 327 (16.0%) required additional variant analysis following short-read WGS analysis, as conclusions about the variant/s sequence, length, or phasing (when relevant) could not be made based on short-read technology alone (Table 1). Therefore, as a second-tier analysis, those cases were additionally sequenced with long-read WGS, generating a double-technology cohort, with 184 cases (i.e., 9.0% of all cases with reportable variants) specifically analyzed to determine the exact length and/or composition of the STR expansions, as well as to achieve accurate haplotyping in the cases of recessive STR conditions.

**Figure 1:**
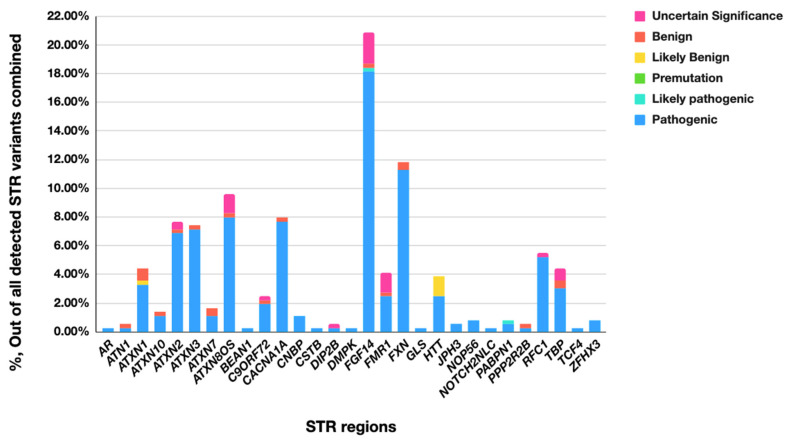
Diagnostic value of STR genotypes using ONT long-read sequencing. Axis X lists STR loci; axis Y indicates % of each category of each STR out of all detected STR variants combined.

The expanded STR variants were reported in 16.6% (338 cases) of all cases with reportable variants. However, only about half of those cases were reportable based on the short-read WGS data alone, while the analysis of the other half required ONT long-reads to determine the expansion range and to identify the exact sequence of the expansion (Table 2). That included all longer expansion variants in the STR regions where the reportable threshold significantly exceeded the length of the short reads, such as longer variants in *FGF14*, *FXN*, *FMR1*, *RFC1*, etc. (Table 2, Figure 1). Long-read sequencing was also required to precisely determine the length of shorter repeat expansions, such as *ATXN2*, *ATXN7,* or *HTT*, in cases where the length of the expansion exceeded 135–140 bp and could not be reliably determined by 150 bp read-based technology (Table 2, Figure 1). It also provided additional information which was useful for variant interpretation, such as the 5mC methylation status (in case of *FMR1* expansions) and allele structure/repeat units composition (such as *FGF14* and *RFC1*, for example), as well as for establishing the pathogenic status of the biallelic expansions for longer repeats when short-read sequencing did not allow for an accurate differentiation between two long pathogenic alleles and one long pathogenic and another long within the subpathogenic range (such as the *RFC1* and *FXN* STR variants).

While 91.2% of the reported STR variants were conclusively classified as either pathogenic or benign following long-read sequencing, 8.8% of variants could not be definitively classified due to repeat expansions falling into previously unannotated length ranges, having unusual repeat unit types or interruptions, or presenting contradictory evidence across the published studies. These variants were classified as variants of uncertain clinical significance (VUS). Therefore, the incorporation of long-read sequencing contributed to an increase in cases with conclusive results including STR expansions by about 40%. Notably, the incorporation of long reads into the diagnosis of other variant types demonstrated more modest improvements (by about 6%), primarily attributed to the lower proportion of variants necessitating additional characterization and the prevalence of variants of uncertain significance (VUSs).

Predictably, the STR variants most frequently detected and characterized with the addition of long-read sequencing were expansions with high reporting threshold and complex expanded region structure within the loci associated with highly prevalent neurological disorders (Figure 1) [12,17,42,43,44]. Of particular interest here was a high fraction of cases with long expansions in the *FGF14* STR region, which was only recently characterized as a common cause of neurological disorder partially overlapping *RFC1*-related phenotypes [43]. This is one of the targets where the collected information contributes to the evolving knowledge base of alleles composition and populational prevalence.

On the other hand, some of the findings represent the variants in well-characterized loci where pathogenic alleles are not much longer than the range precisely detectable with short-read sequencing. A notable example is the *HTT* STR region which, despite a moderate expansion length, frequently required supplementation with long-read sequencing and ultimately yielded a significant fraction of alleles characterized as non-pathogenic (Figure 1). This stems from the close proximity of ranges between normal and abnormal alleles, with a strong association of expansion length with disease penetrance and the age of onset, thus necessitating rigorous verification of the expansion length.

Considering that long-read sequencing generates reads often covering the entire length of the expanded STR region, even for the longest variants, it provides a unique opportunity to analyze the structure of the expanded regions and review somatic mosaic composition (with various repeat units and the exact length of each fragment), where applicable. To fully benefit from this feature, a careful visual inspection of the long-read sequenced STR regions is routinely conducted at Variantyx at the time of clinical interpretation, recording the allele ranges and structures. This visual inspection is aligned with the best practices and guidelines for variant interpretation and reporting in NGS-based clinical genetic testing [23,44,45].

We utilized long-read-based WGS results to characterize the repeat motifs, mosaicism, and number of repeat interruptions to further characterize some of the STR loci with the most frequently detected variants in the movement disorder patient cohort.

We observed that some longer STR variants demonstrated a significant mosaicism of expansion length and composition of the expanded alleles. We were able to detect high variability of the repeat units and motifs that deviate from the frequent repeat composition. The atypical repeat unit and allele composition were especially prominent in the STR regions where the pathogenic repeat units differ from the regular repeat units observed in the unaffected individuals. For example, in the *RFC1* STR region, the typical benign repeat unit is AAAAG, while the most frequent pathogenic repeat unit is AAGGG, with AAAGG and multiple additional repeat units of various pathogenicity encountered on rarer occasions [39,46,47].

**Figure 2:**
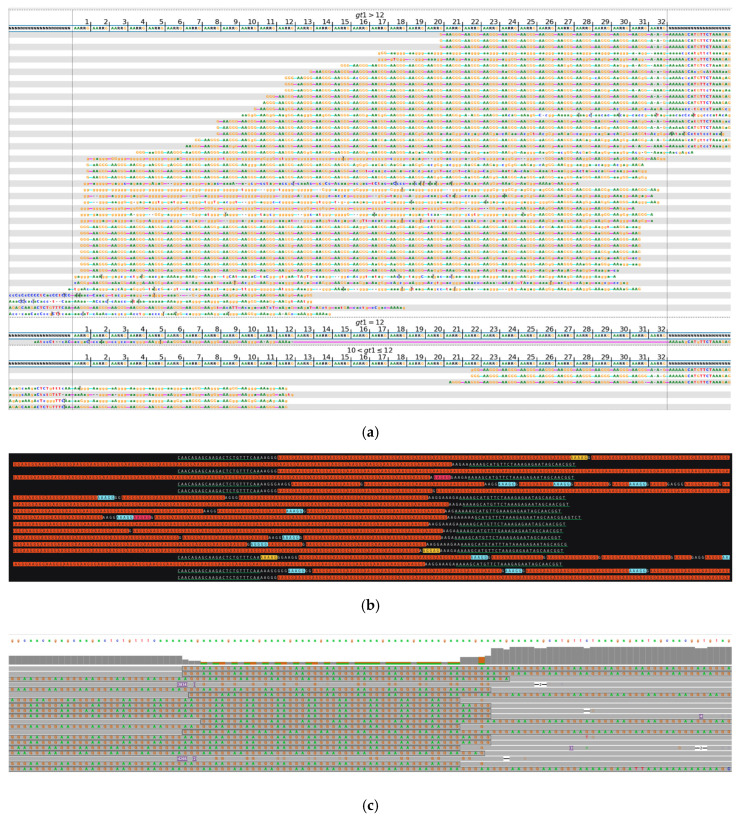
*RFC1*, biallelic expansion. (**a**) Visualization of short-read sequencing results, showing reads flanking the STR regions and those fully contained within the repeat. The bioinformatically predicted genotype based on read statistics is 49/49 * repeats. (**b**) Visualization of ONT long-read sequencing data, depicting a biallelic expansion of >200/>830 repeats, primarily composed of AAGGG pathogenic repeat units with occasional interrupting sequences. Green-underlined sequences highlight the regions flanking the STR repeat. Due to read length limitations, most of the reads are not able to capture both flanking regions of this repeat expansion. Different repeat units are highlighted in different colors: AAGGG (red), AAAGG (blue), AGAAG (yellow). (**c**) Integrative Genomic Viewer (IGV) visualization of ONT long-read sequencing data over the *RFC1* target region. Purple rectangles indicate insertions. * Results of bioinformatic prediction not depicted in the image.

Figure 2 shows a biallelic repeat expansion composed mostly of AAGGG pathogenic repeats as detected by long-read sequencing. In contrast, in the initial analysis of this sample with short-read WGS, where the variant length and composition were inferred from much shorter sequence blocks, an expansion of uncertain length and the presence of pathogenic repeat units were detected, but it was not possible to place the alleles into the pathogenic range and detect the exact structure of the locus.

**Figure 3:**
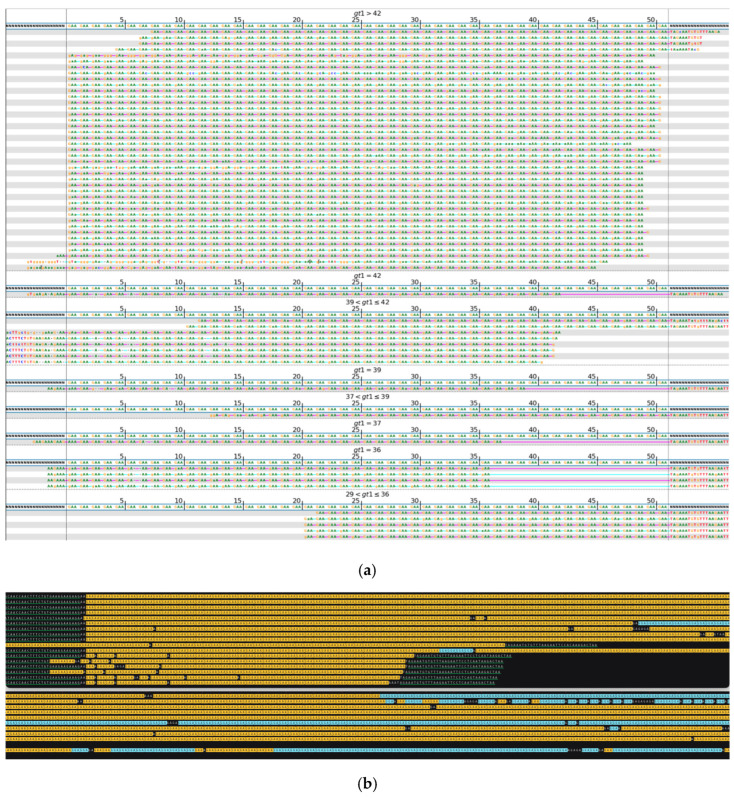
*FGF14*, heterozygous variant. (**a**) Visualization of short-read sequencing results, showing reads flanking the STR regions, as well as those fully contained within the repeat. The bioinformatically predicted genotype based on read statistics is 36/105 * repeats. (**b**) Visualization of the ONT long-read sequencing results, depicting a heterozygous repeat expansion of 41/420–481 repeats. The expansion shows mosaicism in both length and sequence, with some reads incorporating long stretches of GGA repeats in addition to the canonical GAA. Green-underlined sequences highlight the regions flanking the STR repeat. Different repeat units are highlighted in different colors: GAA (yellow), GGA (blue). The sequence shown in the upper panel continues in the lower panel. Due to limitations of the space and the length of the reads, some of them do not have both flanks visible in the included image. * Results of bioinformatic prediction not depicted in the image.

**Figure 4:**
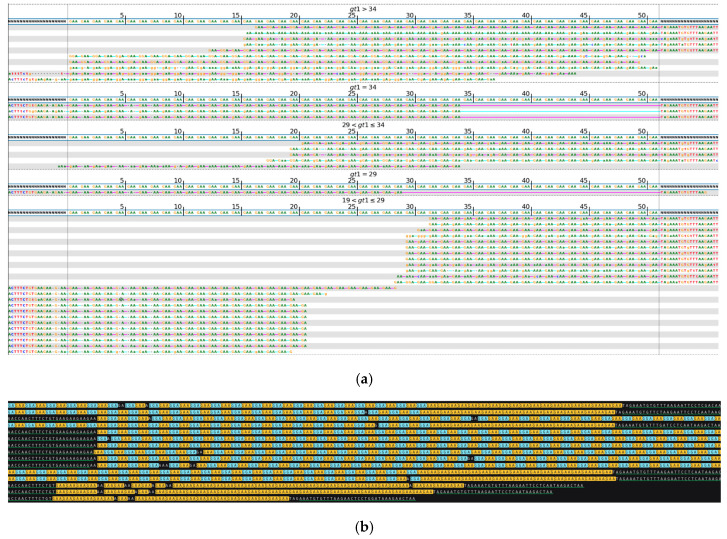
*FGF14*, with a noncanonical GAAGGA repeat unit. (**a**) Visualization of short-read sequencing results showing reads flanking the STR regions as well as those fully contained within the repeat. The bioinformatically predicted genotype based on read statistics is 36/56 * repeats. (**b**) Visualization of the ONT long-read sequencing results, depicting a heterozygous repeat expansion with 35–37/318–342 repeats, where the shorter allele is composed of canonical GAA repeats, while the longer one is predominantly composed of GAAGGA repeats, followed by a short stretch of canonical GAA. Green underlined sequences highlight the regions flanking the STR repeat. Different repeat units are highlighted in different colors: GAA (yellow), GGA (blue). Due to limitations of the space and the length of the reads, some of them do not have both flanks visible in the included image. * Result of bioinformatic prediction, not depicted in the image.

During the analysis of long expansions, we observed not only mosaicism in the repeat length and the location of interruptions but also instances where alleles were predominantly composed of noncanonical repeat units. Such cases are displayed in Figure 3, with a mosaic expanded variant where some of the reads with longer *FGF14* expansions incorporate stretches of GGA repeats alongside the canonical GAA, as well as in Figure 4, with a longer allele composed mostly of GAAGGA 6-nucleotide repeat followed by a short segment of a canonical GAA 3-nucleotide expansion. Previous studies suggest that non-GAA repeats are likely non-pathogenic [21,29].

**Figure 5:**
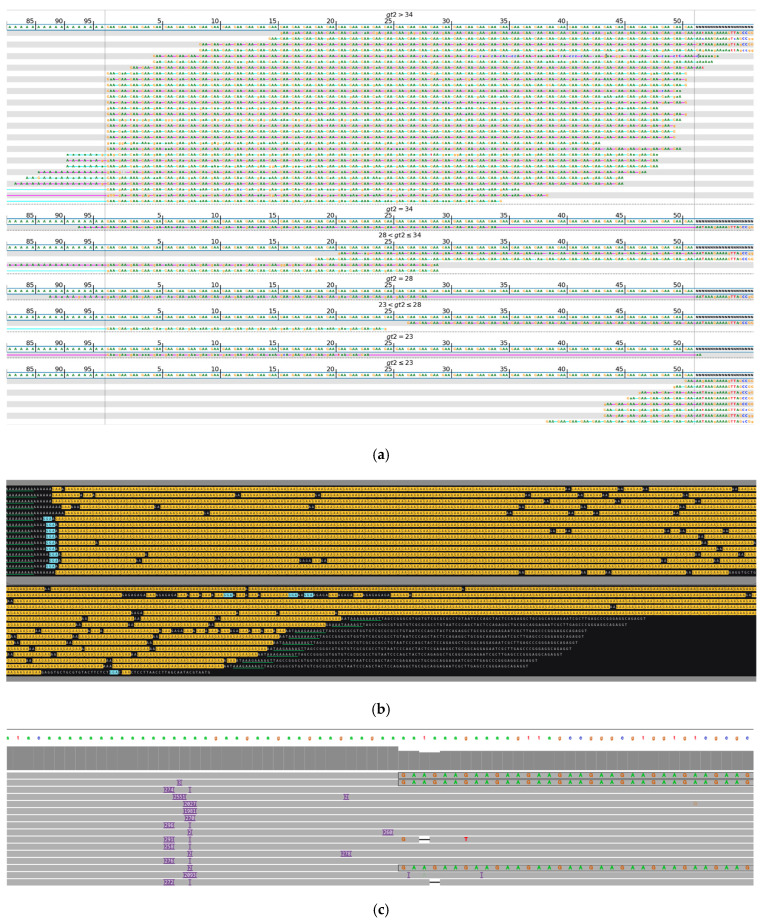
*FXN*, biallelic repeat expansion. (**a**) Visualization of short-read sequencing results, showing reads flanking the STR regions as well as those fully contained within the repeat. The bioinformatically predicted genotype based on the read statistics is 76/76 * repeats. (**b**) Visualization of the ONT long-read sequencing results, depicting a biallelic repeat expansion with one allele of 92–104 repeats and another one of 649–901, composed mainly of GAA units (highlighted in yellow) with occasional GGA interruptions (highlighted in blue). The sequence shown in the upper panel continues in the lower panel. Green-underlined sequences indicate regions flanking the STR repeat. Due to limitations of the space and the length of the reads, some of them do not have both flanks visible in the included image. (**c**) Integrative Genomic Viewer (IGV) visualization of ONT long-read sequencing data over the *FXN* target region. Purple rectangles indicate insertions. * Results of bioinformatic prediction not depicted in the image.

In addition to determining the specific length and structure of the complex STR regions, long-read sequencing offers the advantage of the accurate detection of the length of both alleles in conditions with a recessive inheritance, such as *FXN*. While the expansion of two alleles above the benign range can be easily observed with short-read sequencing, distinguishing between two long pathogenic alleles and a scenario where one is a very long allele while the other is either moderately expanded or falls within the intermediate range can be challenging. Short read-based analysis of long STR expansions typically relies on statistical predictions derived from the counts of reads flanking the repeats and fully encompassed by them. In contrast, long-read sequencing is an invaluable tool in such cases, enabling the direct determination of the full length of each allele. Figure 5 shows a biallelic *FXN* repeat expansion, where short read-based evaluation predicted a 76/76 genotype with two similar length pathogenic alleles composed predominantly of GAA repeats. However, an estimation of the GAA repeat expansion length based on the statistical analysis of reads derived from short-read sequencing cannot be accurate due to the general abundance of unrelated genomic loci composed of GAA repeats in the human genome. The long-read sequencing of this case demonstrated one of the alleles of around 100 repeats, while the other extended beyond 600 repeats. This information is crucial for assessing disease severity and predicting the age of onset.

**Figure 6:**
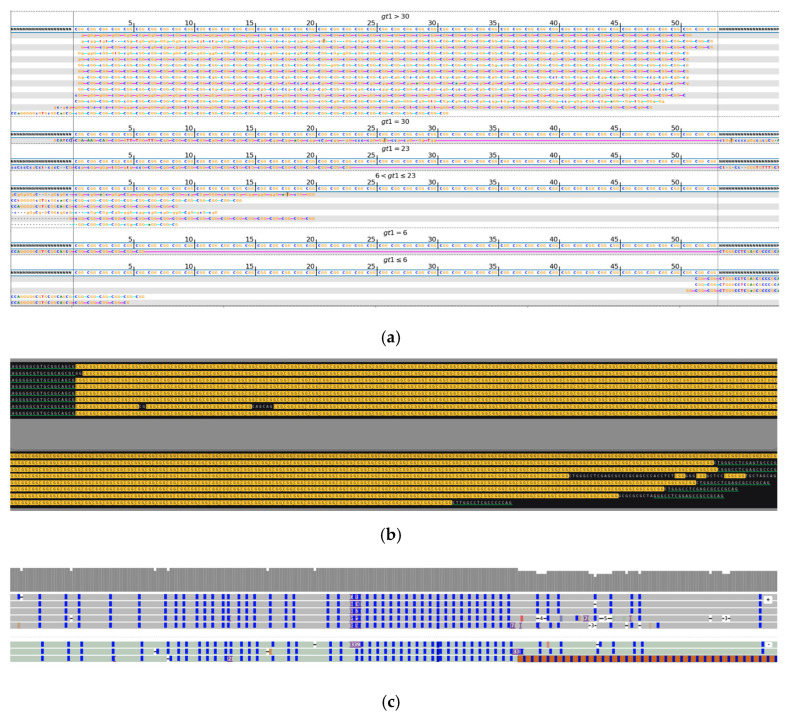
*FMR1*, male, permutation. (**a**) Visualization of short-read sequencing results showing reads flanking the STR regions, as well as those fully contained within the repeat. The bioinformatically predicted genotype based on read statistics is 156 * repeats. (**b**) Visualization of the ONT long-read sequencing results, showing an expansion of 130–132 repeats (highlighted in yellow). Green-underlined sequences indicate regions flanking the STR repeat. The sequence shown in the upper panel continues in the lower panel. Due to limitations of the space and the length of the reads, some of them do not have both flanks visible in the included image. (**c**) Integrative Genomic Viewer (IGV) visualization of the ONT long-read sequencing results with cytosines in CpG context color coded according to their epigenetic status: red for methylated and blue for unmethylated cytosines. No 5mC methylation is observed in this region. * Results of bioinformatic prediction not depicted in the image.

**Figure 7:**
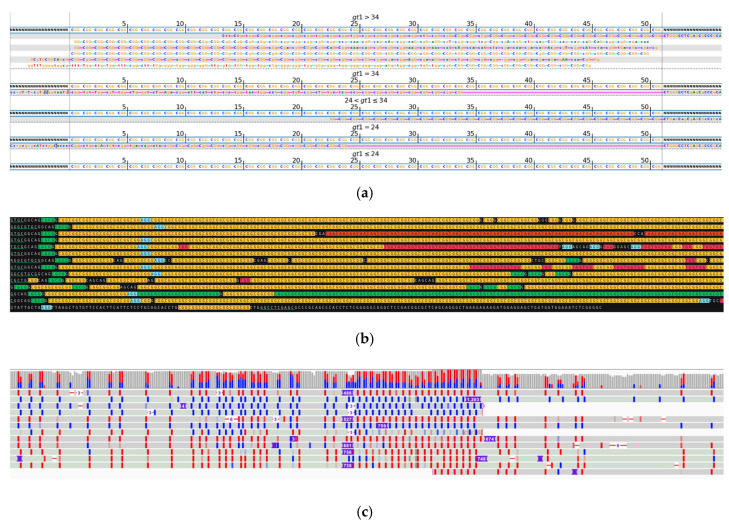
*FMR1* mosaic expansion, male. (**a**) Visualization of short-read sequencing results showing reads flanking the STR regions, as well as those fully contained within the repeat. The bioinformatically predicted genotype based on the read statistics is 81 repeats *. (**b**) Visualization of the ONT long-read sequencing results showing a total expansion length of 270–400 repeats, including 44–396 canonical CGG units and stretches of various noncanonical repeats in some of the reads. Green-underlined sequences indicate regions flanking the STR repeat. Different repeat units are highlighted in different colors: CGG (yellow), TGG (red), AGG (blue), CGCG (green). Due to limitations of the space and the length of the reads, some of them do not have both flanks visible in the included image. (**c**) Integrative Genomic Viewer (IGV) visualization of the ONT long-read sequencing results with cytosines in CpG context color coded according to their epigenetic status—red for methylated and blue for unmethylated cytosines. 5mC methylation is observed on the reads with expanded canonical repeats but only in the absence of long stretches of TGG, AGG, CAA interruptions. * Results of bioinformatic prediction not depicted in the image.

Epigenetic modifications represent another type of information that can be detected by long-read sequencing, contributing valuable diagnostic information when relevant. This is particularly true in cases of *FMR1* expansions, where pathogenicity has been associated with 5mC methylation in CpG context. In specimens with mosaic repeat expansions and/or interruptions in the long repeat regions, the simultaneous detection of 5mC methylation or lack thereof offers a means to better evaluate the presumed level of pathogenicity [48,49]. Figure 6 shows *FMR1* region in a male with premutation, where no 5mC methylation in the CpG context is observed in any of the reads. In contrast, Figure 7 illustrates a mosaic pathogenic *FMR1* expansion in a male, where longer stretches of interrupting repeats are present in some of the reads, and 5mC methylation in the CpG context is observed only on the expanded repeats without long TGG, AGG, CAA interruptions.

**Figure 8:**
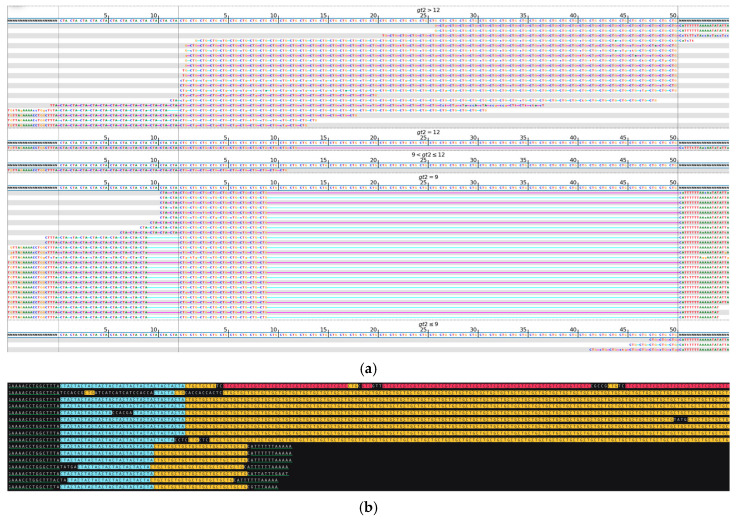
*ATXN8OS*, heterozygous repeat expansion, complex region with CTA-CTG structure. (**a**) Visualization of short-read sequencing results showing flanking the STR regions, as well as those and reads fully contained within the repeat. The bioinformatically predicted genotype based on the read statistics is 9 + 9/12 + 79 * repeats (CAT + CTG/CTA + CTG complex region). (**b**) Visualization of the ONT long-reads sequencing results showing total expansion length and structure—9 CAT + 9 CTG repeats (shorter, normal allele) and 12 CTA + 83–84 CTG repeats (expanded allele). Green-underlined sequences indicate regions flanking the STR repeat. Different repeat units are highlighted in different colors: CTG (yellow), CTA (blue), GTC (red). * Results of bioinformatic prediction not depicted in the image.

Figure 8 illustrates the detection of the length and structure of complex *ATXN8OS* repeat locus. Both the normal and expanded alleles have similar patterns of repeat units, where a segment consisting of CTA repeats is followed by a segment consisting of CTG repeats, while the length of each segment varies between the alleles. Some of the traditional STR detection techniques report such complex regions only in terms of their length, while sequencing allows to acquire more comprehensive information about the region structure as well as and expansion length.

## 3. Discussion

Application of a two-tiered sequencing approach allows Variantyx to fully leverage the advantages of long-read sequencing technology in routine clinical genetic testing, while keeping the laboratory and analysis processes as simple as possible and the costs reasonably low. Both short- and long-read WGS provide comprehensive genomic coverage, unlike the targeted tests that currently prevail in the clinical genetic testing landscape. This approach allows for the rapid incorporation of newly discovered disease-causing STR expansions into clinical testing. For example, we were able to report our first pathogenic *ZFHX3* expansion to a patient only several weeks after its association with SCA4 was published [50]. A clinical validation study performed by the laboratory revealed close to 100% sensitivity for most variant types [51,52]. This study included over 200 positive STR cases across various genes and repeat units. Comparing the diagnostic yield resulting from incorporating Oxford Nanopore Technologies (ONT) long-read sequencing in the analysis of different variant types, we observed a significantly higher impact in detection and characterization of STR expansions compared to other variant categories. Although long-read sequencing can benefit the detection of structural variants, variants with epigenetic changes and, to some extent, small sequence changes, this approach is particularly useful for the comprehensive analysis and interpretation of long and complex STR expansion variants.

The precise detection of STR expansion is particularly critical for patients with movement disorders and dementia. In the cohort of 2689 patient cases analyzed in this study, reportable variants were detected in 2038 cases (75.8%), including secondary and incidental findings, among which 1166 cases (43.4%) carried positive diagnostic results, examples of which are presented in Figure 2, Figure 3, Figure 4, Figure 5, Figure 6, Figure 7 and Figure 8. Among the 2038 reportable cases, 338 included relevant STR variants, approximately half of which required the use of ONT long-read sequencing.

While long-read data provide more information on the size and composition of repeat expansions than any other technique used in clinical genetic testing, the differences in the observed long-read sequencing results as compared to the laboratories utilizing other techniques may present challenges in clinical interpretation.

The existing interpretation guidelines and the majority of publications based on the results obtained with other approaches do not take into account the somatic mosaicism that is often observed in patients, particularly in the STRs marked in color in Table 2 [12,29,30,33,34,35,36,37,38,39,40]. For instance, in *RFC1* expansions, the pathogenic repeat unit is often present only at the edges of the expansion, while the bulk of the expanded sequence is composed of different repeat units or combinations of repeated and non-repeated sequences (unpublished data to be summarized in a future publication). Most other detection techniques, which rely on the amplification or hybridization of stretches of pathogenic repeat units, will incorrectly assume in such cases that the entire expansion consists solely of the pathogenic repeat units. Consequently, in statistical studies that form the basis for interpretation guidelines, the defined expansion ranges associated with the pathogenicity of the observed repeat units differ from those detected with long reads. The direct application of these guidelines to the clinical interpretation of ONT long-read sequencing-derived results could lead to incorrect reporting.

Until further cohort studies utilizing this technology are conducted [5,53] and technology-specific guidelines are developed, results for certain patient cases may need to be reported as uncertain. The capacity to characterize unusual sequences, particularly in cases of mosaic expansions which cannot be accurately assessed without a single long molecule approach, increases the value of long-read sequencing by expanding the knowledge base during routine clinical analysis. The current standards in the clinical genetic testing of STR expansion variants are mostly geared toward the detection of STR expansion length and the inspection of known motifs, therefore potentially failing to identify the unusual variations affecting expansion pathogenicity and stability [54]. For example, minor interruptions in *FXN* repeat expansion encountered in more than 70% of Friedreich’s Ataxia patients are not assessed by some of the currently offered genetic tests [40,55]. Recent updates in the ACMG technical standards recognize the detection and reporting challenges and advise to use caution when the reliability of the methodology might be affected by interruptions (for example, repeat-based PCR for the detection of CTG repeats such as *DMPK* where interruptions might increase the GC content, causing allele bias [56]). Another example that presents a challenge to traditional testing technologies is a multitude of various unusual repeat motifs in the *RFC1* STR region, which may be associated with an atypical phenotypic presentation in patients with cerebellar ataxia and have the potential to remain undiagnosed unless the appropriate long-range testing methodologies are employed [57,58]. At Variantyx, we are working on building a population cohort that will help the clinical community to establish updated guidelines for the interpretation of long-read sequencing data. Our database, which currently includes tens of thousands of short-read WGSs and thousands of ONT long-read WGS datasets from patients with a variety of phenotypes, is rapidly growing. Due to patient privacy considerations, we will not be able to make the raw sequencing data publicly accessible; however, we are planning to share our summarized observations in the near future. These data were generated using short-read WGS as a primary methodology, supplemented with long-read WGS when necessary.

Another direction of future development would be switching entirely to long-read-based WGS, which would simplify the wet bench and bioinformatic analysis processes and potentially further improve the detection power of the genetic testing. At the current stage in the development of long-read technology, however, this approach is not yet feasible for large-scale routine diagnostic testing. Currently, the two main long-read-based NGS technologies are ones developed by Pacific Biosciences (PacBio) and by Oxford Nanopore Technologies (ONT) [59,60,61]. While PacBio HiFi technology generates long-read data accuracy nearly comparable to the industry-standard short-read sequencing for small sequence changes and copy number variants, the maximum length limitations render it unsuitable for the detection of extremely long variants. The current generation of PacBio sequencing machines is also low throughput, relative to the leading short-read sequencing solutions. Conversely, ONT long reads are highly useful in evaluating the copy number and structural variants of various sizes but less accurate in detecting small sequence changes compared to short-read sequencing.

The main constraint of long-read sequencing technologies that are currently commercially available is their relatively high cost, which at this time does not allow them to be used as a standalone solution for standard first-line diagnostic and screening testing. Therefore, these NGS technologies, together with a multitude of additional approaches (such as Illumina long reads [62]), still require further development before potentially serving as standalone comprehensive clinical testing tools suitable for any types of genetic disorders. Long-read-based sequencing technologies are rapidly evolving, and we expect that, in several years, these constraints will be resolved. Until then, a combinatorial approach in which long-read sequencing is used to supplement the short-read WGS (when needed) and which can significantly enhance clinical utility of the test is well suited for clinical genetic testing. In this study, we demonstrate that this is particularly true for diagnostics of movement disorders and dementia, which are often caused by genetic variants that are challenging to analyze using short-read sequencing alone, such as STR expansions.

## 4. Materials and Methods

### 4.1. Samples Selection

The study cohort consisted of patients with ataxia/dementia phenotypes, according to the standard HPO classification, and was analyzed at the Variantyx genetic testing laboratory (Framingham, MA, USA) over a study period of 24 months (2023 and 2024). The inclusion criteria were not limited to any gender, age, or ethnicity category of participants. The cohort was composed of all the specimens submitted to the Variantyx laboratory within the study period for germline genetic testing for neurological disorders and/or with neurological disorders phenotypes listed in the accompanying medical documentation.

All participants signed an informed consent prior to inclusion into the current study and all samples were deidentified.

Specimen collection was performed with Lavender K2-EDTA Blood Collection Tubes (367861, PulmoLab, Northridge, CA, USA) and saliva collection tubes (OGD-500 and OCR−100, DNA Genotek, Ottawa, Ontario, Canada) and extracted at Variantyx using the QIAsymphony, Qiagen EZ2, or QIAamp kit (cat #51106, QIAGEN, Venlo, The Netherlands) or delivered as pre-extracted gDNA from the submitting laboratories as arranged by the medical providers ordering the genetic testing.

### 4.2. Library Preparation and Illumina Short-Read Sequencing

A total of 300 ng of gDNA was used for library preparation utilizing the Illumina WGS PCR-Free Tagmentation protocol with multiplex ligation sequencing kits using Standard Workflow reagents (Cat# 20041794/20041795, 20,028,312 Illumina Inc., San Diego, CA, USA). The samples were sequenced using NovaSeq 6000 and NovaSeq X Plus sequencers (Illumina Inc., San Diego, CA, USA) with S4 and 10B/25B flow cells.

### 4.3. Bioinformatics and Analytical Approach Illumina Short Reads

The sequencing data were analyzed using the Variantyx Genomic Intelligence bioinformatic pipeline, combining publicly available and in-house developed tools for the detection of Small Sequence Changes (SSCs), Structural Variants including Copy Number Variants (SVs and CNVs), Mitochondrial SSCs with Heteroplasmy, Uniparental Disomy (UPD), and SMN1/2 Copy Numbers, as well as for the detection of STR variants and the prediction of the length of long expanded alleles [51]. Genetic variants as detected by the pipeline were visualized, analyzed, and reported with the in-house developed Diagnostic Console.

### 4.4. Library Preparation and ONT Long-Read Sequencing

A total of 1500 ng of gDNA was sheared with the FastPrep−96™ High-Throughput Bead Beating Grinder and Lysis System (MP Biomedicals, Irvine, CA, USA, Cat# SKU 116010500) at 1800 rpm for 3 min. The resulting DNA fragments were used for library preparation, utilizing the ONT Ligation sequencing gDNA protocol with multiplex ligation sequencing kits V11/14 (Cat# SQK-MLK111.96-XL/SQK-MLK114.96-XL, ONT, Oxford, UK) with incorporated native barcoding and low molecular weight fragment elimination buffer. Samples were sequenced using PromethION P24 ONT device (ONT, Oxford, UK) with R9.4/R10.4.1 flow cells (Cat# FLO-PRO002M/FLO-PRO114M, ONT, Oxford, UK).

### 4.5. Bioinformatics and Analytical Approach to ONT Long-Reads

Basecalling on high-accuracy settings (HAC) and demultiplexing were performed in parallel with sequencing using the MinKNOW v.23 software (ONT, Oxford, UK) integrated with PromethION P−24 sequencing device. The basecalling process also included the identification of the epigenetic modifications 5mC/5hmC in the CpG context. The HAC settings were selected based on the recommendations of the manufacturer as a high-quality approach compatible with real-time basecalling and recommended for projects focusing on variant analysis.

The acquired reads were processed with Variantyx proprietary Genomic Intelligence platform (https://www.variantyx.com/, accessed on 10 February 2025), generating long-read alignment with Minimap2 (v.2.23 https://github.com/lh3/minimap2, accessed on 1 December 2022). Genome assembly hg38 [63] was used as a reference.

Aligned reads were visualized with the Integrative Genomics Viewer (IGV, v3.0.2, https://github.com/igvteam/igv.js, accessed on 1 October 2024) and with the in-house developed diagnostic console. For short tandem repeat analysis, the results were manually inspected, registering both the regular and pathogenic repeat units (when applicable), as well as the presence of interrupting sequences. Definitions of the repeat regions, along with annotations of the normal and expanded repeat ranges, were retrieved from the Genome Aggregation Database [64], the Database of Short Tandem Repeats [65], and the STRchive [10].

In addition to the detection of genetic variants, 5mC methylation in the CpG context in the regions of interest was recorded when relevant.

## Figures and Tables

**Table 1:** Distribution of cases with reportable variants by sequencing technology and types of reported variants.

	No Long-Read Sequencing Required	Long-Read Sequencing Required	Total
Cases with STR expansion	184 (9.0%)	154 (7.6%)	338 (16.6%)
Cases with Other variant types (No STR)	1527 (74.9%)	173 (8.5%)	1700 (83.4%)
Total	1711 (84.0%)	327 (16.0%)	2038 (100.00%)

**Table 2:** STR variant ranges and detection technology used for reporting in the movement disorders and dementia cohort *.

	Ranges						Reported Variants		
STR Locus	Normal Range	Mutable Normal	Intermediate/Uncertain Range	Reduced Penetrance Range	Pathogenic Threshold	Repeat Unit Length	No Long-Read Sequencing	Long-Read Sequencing	Total
*AR*	<34		35	36–37	38	3	1	0	1
*ATN1*	6–35			36–47	48	3	1	1	2
*ATXN1*	6–35 (36–44 **)	36–38			39 (46 *)	3	11	5	16
*ATXN10*	10–32		33–280	280–800	800	5	3	2	5
*ATXN2*	<30		30–32 ***	33–34	35	3	23	5	28
*ATXN3*	44		45–49	50–55	56	3	18	8	26
*ATXN7*	27	28–33		34–36	37	3	3	3	6
*ATXN8OS*	15–50		51–53	54 ^	54 ^	3	23	11	34
*BEAN1* ^^	-			-	110	5	1	0	1
*C9ORF72*	≤24		25–60	24–60	61	6	6	3	9
*CACNA1A*	18		19	19–19	20	3	25	3	28
*CNBP*	26		27–74		75	4	1	3	4
*CSTB*	2–3	12–17			30	12	0	1	1
*DIP2B*	6–23			139–206	250	3	0	1	1
*DMPK*	5–34	35–49			50	3	1	0	1
*FGF14*				180–319	320	3	22	54	76
*FMR1*	5–44		45–54	55–200 ^^^	201	3	6	10	16
*FXN*	5–33		34–65		66	3	29	13	42
*GLS*	5–38			-	680	3	0	1	1
*HTT*	26	27–35		36–39	40	3	2	7	9
*JPH3*	28			29–39	40	3	0	2	2
*NOP56*	3–14			15–649	650	6	0	3	3
*NOTCH2NLC*	≤40		41–59		60	3	0	1	1
*PABPN1*	10			11–11 homozygous	11–18	3	2	1	3
*PPP2R2B*	7–32			40–50	51	3	2	0	2
*RFC1*				11–200	400	5	5	15	20
*TBP*	25–40			41–48	49	3	12	3	15
*TCF4*	40			40–50	51	3	1	0	1
*ZFHX3*	31		31–41		42	3	1	2	3

* Many patients included in the cohort had complex phenotypes with partial overlap with movement disorders/dementia symptoms. ** Interrupted range. *** Range of association with amyotrophic lateral sclerosis. ^ Full penetrance alleles are not recognized in this STR. ^^ *BEAN1* expansions cannot be detected using short-read WGS and require primary variant calling using ONT data. Thus, diagnostic tests in which analysis of this STR is included do not utilize the two-tier sequencing approach. Instead, samples are sequenced with both short- and long-read-based WGS simultaneously. Such tests are thus excluded from the presented patient cohort, and the only positive *BEAN1* case included in this study underwent ONT WGS sequencing to evaluate a different STR expansion that was eventually confirmed to be negative. ^^^ Premutation FXTAS/FXPOI. STR regions with somatic mosaicism shown in purple font.

## Data Availability

The aggregated and processed data supporting the findings of this study are available from the corresponding author upon request. The raw sequencing data cannot be made publicly available as they contain sensitive patient information protected under privacy laws and informed consent agreements.

## Abbreviations

NGS	Next-Generation Sequencing
WGS	Whole-Genome Sequencing
STR	Short Tandem Repeat
VNTR	Variable Number Tandem Repeat
ONT	Oxford Nanopore Technologies
HPO	Human Phenotype Ontology
gDNA	Genomic DNA
SSC	Small Sequence Change
SV	Structural Variant
CNV	Copy Number Variant
UPD	Uniparental Disomy
HAC	High-Accuracy Settings
IGV	Integrative Genomics Viewer
VUS	Variant of Uncertain Significance
SCA	Spinocerebellar Ataxia
FXTAS	Fragile X-Associated Tremor/Ataxia Syndrome
FXPOI	Fragile X-Associated Primary Ovarian Insufficiency
